# Trends and Correlates of Breakthrough Infections With SARS-CoV-2

**DOI:** 10.3389/fpubh.2022.856532

**Published:** 2022-05-10

**Authors:** Jose-Miguel Yamal, Savitri Appana, Mengxi Wang, Luis Leon-Novelo, Eric Bakota, Yuanqing Ye, Shreela Sharma, Alanna C. Morrison, Dritana Marko, Stephen H. Linder, Alison Rector, Katelyn K. Jetelina, Eric Boerwinkle, Marcia de Oliveira Otto

**Affiliations:** ^1^Department of Biostatistics and Data Science, The University of Texas Health Science Center at Houston (UTHealth) School of Public Health, Houston, TX, United States; ^2^The Office of Science, Surveillance, and Technology, Harris County Public Health, Houston, TX, United States; ^3^Department of Epidemiology, Human Genetics and Environmental Sciences, The University of Texas Health Science Center at Houston (UTHealth) School of Public Health, Houston, TX, United States; ^4^Department of Management, Policy and Community Health, Institute for Health Policy, The University of Texas Health Science Center at Houston (UTHealth) School of Public Health, San Antonio, TX, United States; ^5^Department of Management, Policy and Community Health, Institute for Health Policy, The University of Texas Health Science Center at Houston (UTHealth) School of Public Health, Houston, TX, United States; ^6^Department of Epidemiology, Human Genetics and Environmental Sciences, The University of Texas Health Science Center at Houston (UTHealth) School of Public Health, Dallas, TX, United States

**Keywords:** COVID-19, COVID-19 vaccine, breakthrough infection, delta variant strain, COVID-19 vaccine efficacy

## Abstract

The severe acute respiratory syndrome coronavirus 2 (SARS-CoV-2) delta variant has been hypothesized to decrease the efficacy of COVID-19 vaccines. Factors associated with infections with SARS-CoV-2 after vaccination are unknown. In this observational cohort study, we examined two groups in Harris County, Texas: (1) individuals with positive Nucleic Acid Amplification test between 12/14/2020 and 9/30/2021 and (2) the subset of individuals fully vaccinated in the same time period. Infected individuals were classified as a breakthrough if their infection occurred 14 days after their vaccination had been completed. Among fully vaccinated individuals, demographic and vaccine factors associated with breakthrough infections were assessed. Of 146,731 positive SARS-CoV-2 tests, 7.5% were breakthrough infections. Correlates of breakthrough infection included young adult age, female, White race, and receiving the Janssen vaccine, after adjustments including the amount of community spread at the time of infection. Vaccines remained effective in decreasing the probability of testing positive for SARS-CoV-2. The data indicate that increased vaccine booster uptake would help decrease new infections.

## Introduction

Currently there is one coronavirus disease (COVID-19) vaccine that has received approval from the Food and Drug Administration in the United States and two with Emergency Use Authorization. These three vaccines were found to substantially reduce the risk of developing symptomatic illness or severe outcomes from COVID-19 in randomized clinical trials (1–3) but the risk of infection, including asymptomatic or mild cases, has not been studied extensively. Several studies have focused on the growing number of breakthrough infections among fully immunized individuals (4). However, there is limited evidence of whether the rise in the number of breakthrough infections may be explained by the rapidly growing number of vaccinated individuals at risk of infection and an increase in infections in the community. Additionally, there are limited data on whether any increase in the percentage of breakthrough cases among all new infections may be a result of the delta variant or waning vaccine effectiveness. Finally, factors associated with the likelihood of breakthrough infections are not well-understood.

To address these key gaps in knowledge, we evaluated trends in breakthrough cases and related socio-demographic factors in a community-based observational study including SARS-CoV-2 case investigation and COVID-19 vaccination data from the Harris County Public Health (HCPH) Department.

## Materials and Methods

### Data Source

Harris County is the third largest county in the U.S., with nearly 4.7 million residents of diverse racial and ethnic backgrounds. Data from HCPH included information on residents of Harris County jurisdiction area (2.4 million residents and over 30 municipalities but not including the city of Houston) who tested positive for SARS-CoV-2 (case investigation data), as well as their vaccination records according to the Texas Department of State Health Services Immunization Registry (ImmTrac2). In this analysis, individuals who had their first positive Nucleic Acid Amplification test (NAAT, such as Reverse Transcriptase Polymerase Chain Reaction [RT-PCR]) between December 14, 2020 (the start date of vaccination efforts in Harris County) and September 30, 2021 were linked with ImmTrac2 records using name and date of birth. Children below 12 years of age were excluded from the analysis because they were not eligible for COVID-19 vaccinations during this time period.

COVID-19-related deaths were obtained from the Harris County Institute of Forensic Sciences and Texas Department of State Health Services. Demographic characteristics (race/ethnicity, age, and gender) were obtained from COVID-19 vaccination records and supplemented with the case database information when missing. The University of Texas Health Science Center at Houston (UTHealth) School of Public Health has a data use agreement with Harris County Public Health to share protected health information data and have been collaborating since July 2020 on the pandemic response. The study protocol was reviewed and approved by the committees for the protection of human subjects of University of Texas Health Science Center at Houston. Statistical analyses were conducted using de-identified data.

### Study Design

We used a combination of two observational cohort designs, where one cohort was the population of all Harris County (excluding City of Houston) residents that tested positive, regardless of their vaccination status. Successful linking with vaccination records was used to identify the subset that were vaccinated and we assumed that the rest of the infections were in unvaccinated people. The second observational cohort used vaccination records to identify those that were fully vaccinated and used unobtrusive identification of any subsequent positive tests in this cohort.

### Definition of Breakthrough Cases

Individuals were classified into (1) breakthrough cases if the first positive NAAT test occurred after being fully vaccinated (≥14 days post vaccination series completed); (2) partial breakthrough if the first positive NAAT test occurred after the first vaccine dose and prior to being fully vaccinated (days 0–13 after series completion); and (3) unvaccinated if the positive NAAT test occurred prior to first vaccination dose or the individual was not matched between the cases and ImmTrac2 databases. This definition includes all recorded breakthrough infections and is broader than the current CDC definition of breakthrough cases since they currently monitor only hospitalized or fatal breakthroughs. Fully vaccinated was defined as 14 days after one dose of Janssen (Ad26.COV2.S) (1) or the second dose of either Moderna (mRNA-1273) (2) or Pfizer/BioNTech (BNT162b2) (3) COVID-19 vaccines. Individuals who received additional doses after completing a vaccine series were classified based on their initial vaccine type received (e.g. if an individual received a Janssen dose and subsequently received a Moderna dose after two weeks, they would be categorized into the Janssen group + booster). We estimated the proportion of the new infections and deaths that were breakthrough, partial breakthrough, and unvaccinated.

### Statistical Analysis

Descriptive statistics of demographic characteristics and vaccine manufacturer were analyzed by the three breakthrough status groups. Univariate analysis was conducted comparing breakthrough to non-breakthrough infections (combining partial breakthrough and unvaccinated groups) using Wilcoxon rank sum, Pearson's Chi-squared, or Fisher's exact test.

To evaluate whether the increases in breakthrough infections could be influenced by heterogeneity in length of time since full immunization status, incidence density was estimated as the number of breakthrough cases divided by the total person-days since fully vaccinated (5). The incidence density was estimated overall and by subgroups. Poisson regression was used to assess the association of factors with breakthrough counts, with the log of total person-days and log of the average of the daily new infections the month of breakthrough infection (as a measure of community spread) as two offsets, including an indicator for the time period June 1, 20201–September 30, 2021 (compared to December 14, 2020–May 31, 2021) of when each individual was fully vaccinated, and the R(t) estimate of infectiousness dichotomized as >1 (as a measure of acceleration) vs. <1. For univariate Poisson models, only the total person-days was used as the offset. We tested interactions between the time indicator and each time period factor separately, and also fit Poisson models in the two time periods using two additional models. These time periods were chosen because the B.1.617.2 (delta) variant began to be detected in CDC analyses in 15% of samples at the beginning of June and in 82% of samples by the end of July in central southern US states, and in 94% of cases throughout the metropolitan Houston area (6, 7). Additionally, the 3rd wave of new cases in Texas spanned the first period and the 4th wave began the first week of July 2021 and was ongoing in August. U.S. Census population estimates were used to estimate the unvaccinated population (8).

For the subset of fully vaccinated individuals, time since being fully vaccinated until a breakthrough case was analyzed using Cox Proportional Hazards regression, with the last date of the data as the time of censoring for those with no breakthrough infection. Individuals that were indicated to have received the Janssen vaccine before the approval date were excluded (*n* = 40).

The percentage of breakthrough infections is expected to increase as the number of fully vaccinated people increases in the population. To differentiate the expected increase in this percentage vs. effectiveness differences (for example due to waning effectiveness or novel variant strains), we estimated the relative risk of infection. The probability of having a positive SARS-CoV-2 test given being fully vaccinated was estimated using Bayes formula. Let *D* denote having a positive test and *FV* denote the fully vaccinated group and $\bar{FV}$ denote those that are not fully vaccinated (including partially vaccinated). Then the conditional probability of having a positive test given being fully vaccinated is estimated as Pr(*D*|*FV*) = Pr(*FV*|*D*) *Pr*(*D*)/Pr(*FV*) = *Pr*(*FV*|*D*) *N*_*D*_/*N*_*FV*_, where *N*_*D*_ is the number of positive tests and *N*_*FV*_ is the number of individuals fully vaccinated at risk of having a breakthrough infection (not already having had a previous breakthrough case). Similarly, $\mathrm{Pr}\left(D|\bar{FV}\right)=\frac{Pr\left(\bar{FV}|D\right)\;N_{D}}{N_{\bar{FV}}}$. The relative risk is then given by $RR=\mathrm{Pr}\left(D|\bar{FV}\right)/Pr\left(D|FV\right)=\frac{\mathrm{Pr}\left(\bar{FV}|D\;\right)N_{FV}}{\left(Pr\left(FV|D\right)\;N_{\bar{FV}}\right)}$.

Boosters were considered as receiving any additional COVID-19 vaccine at least 15 days after completing the initial vaccine series. People who had an infection between the first dose and the booster were grouped with those without a booster since they had not received the booster prior to the breakthrough infection. The analyses were performed with R software, version 4.1.1 (R Foundation for Statistical Computing), and SAS software, version 9.4.

## Results

Between December 14, 2020 and September 30, 2021, 1,272,611 (45%) Harris County jurisdiction residents (living outside the City of Houston) were fully vaccinated and 146,731 individuals tested positive for SARS-CoV-2. The median (interquartile range [IQR]) age at the time of infection was 37 years (25 to 51), and 54% of individuals were female. A greater proportion of those testing positive were Hispanic (46%), followed by Whites (27%), Blacks (14%), and Asians (5%). Of residents that had received a vaccine dose, the majority received Pfizer/BioNTech vaccines (60%), followed by Moderna (32%), and Janssen (8%). Janssen doses were first approved for emergency use 3 months after Pfizer/BioNTech and Moderna vaccines.

Table 1 describes the distribution of new SARS-CoV-2 infections among breakthrough categories. Among new infections, 129,912 (89%) occurred among unvaccinated residents, 5,774 (4%) among partially vaccinated, and 11,045 (8%) among fully vaccinated individuals. The median (IQR) number of days between completing the vaccine series and having a breakthrough infection during the 275 days of follow-up was 133 days (IQR 101 to 164).

**Table 1:** Descriptive statistics of 146,731 individuals 12 years or older in Harris County (excluding City of Houston jurisdiction) with a positive SARS-CoV-2 molecular test between 12/14/2020 and 09/30/2021 and their vaccination category^a^.

	**No breakthrough**			**Breakthrough**
	**Unvaccinated at time of infection, *N* = 129,912 (89%)**	**Partial breakthrough post 1st dose, *N* = 5,247 (3.6%)**	**Partial breakthrough post vaccine series complete, *N* = 527 (0.4%)**	**Post fully vaccinated breakthrough, *N* = 11,045 (7.5%)**
Age—year				
Median (IQR)	36 (24–50)	43 (29–58)	46 (34–60)	46 (34–60)
Distribution—no. (%)				
12–19	20,832 (16%)	645 (12%)	59 (11%)	744 (6.7%)
20–39	51,979 (40%)	1,622 (31%)	135 (26%)	3,320 (30%)
40–59	40,203 (31%)	1,764 (34%)	197 (37%)	4,125 (37%)
60–79	14,788 (11%)	996 (19%)	108 (20%)	2,506 (23%)
80+	2,110 (1.6%)	220 (4.2%)	28 (5.3%)	350 (3.2%)
Gender—no. (%)				
Female	62,461 (53%)	2,969 (57%)	302 (57%)	6,380 (58%)
Male	54,940 (47%)	2,274 (43%)	225 (43%)	4,623 (42%)
Other/Unknown	12,511	4	0	42
Race/ethnicity—no. (%)				
Asian American/Pacific Islander	4,916 (4.5%)	270 (5.2%)	24 (4.6%)	796 (7.3%)
Black	15,028 (14%)	689 (13%)	80 (15%)	1,282 (12%)
Hispanic/Latino	52,233 (48%)	1,938 (37%)	152 (29%)	3,849 (35%)
White	28,520 (26%)	1,502 (29%)	169 (32%)	3,932 (36%)
Other^b^	9,109 (8.3%)	788 (15%)	97 (19%)	1,101 (10%)
Unknown	20,106	60	5	85
Median days to infection (IQR)				
After first dose	NA	13 (7, 27)	28 (23, 32)	153 (122, 185)
After vaccine series completed	NA	NA	6 (3, 9)	133 (101, 164)
Vaccine trade name—no. (%)				
Janssen	2,254 (5.6%)	0 (0%)	92 (17%)	1,397 (13%)
Moderna	11,359 (28%)	1,533 (29%)	91 (17%)	2,887 (26%)
Pfizer	26,799 (66%)	3,714 (71%)	344 (65%)	6,761 (61%)
Unknown	89,500	0	0	0

a *All P values (omitted from the table) comparing breakthrough vs. other groups combined <0.001. Wilcoxon rank sum test; Pearson's Chi-squared test*.

b *American Indian/Alaskan Native and Multi-racial groups were included in the “other” category. Individuals with unknown race/ethnicity were removed from the statistical models*.

Compared to infections among unvaccinated or partially vaccinated residents, breakthrough cases were older (median 46 years breakthrough vs. 37 years unvaccinated/partially vaccinated), predominantly female (58% breakthrough vs. 53% unvaccinated/partially vaccinated), White (36% vaccinated vs. 26% unvaccinated/partially vaccinated), and more likely to have received the Janssen vaccine (13% vaccinated vs. 5% unvaccinated/partially vaccinated).

### Change in Percentage of Breakthrough Cases and Deaths Over Time

**Figure 1:**
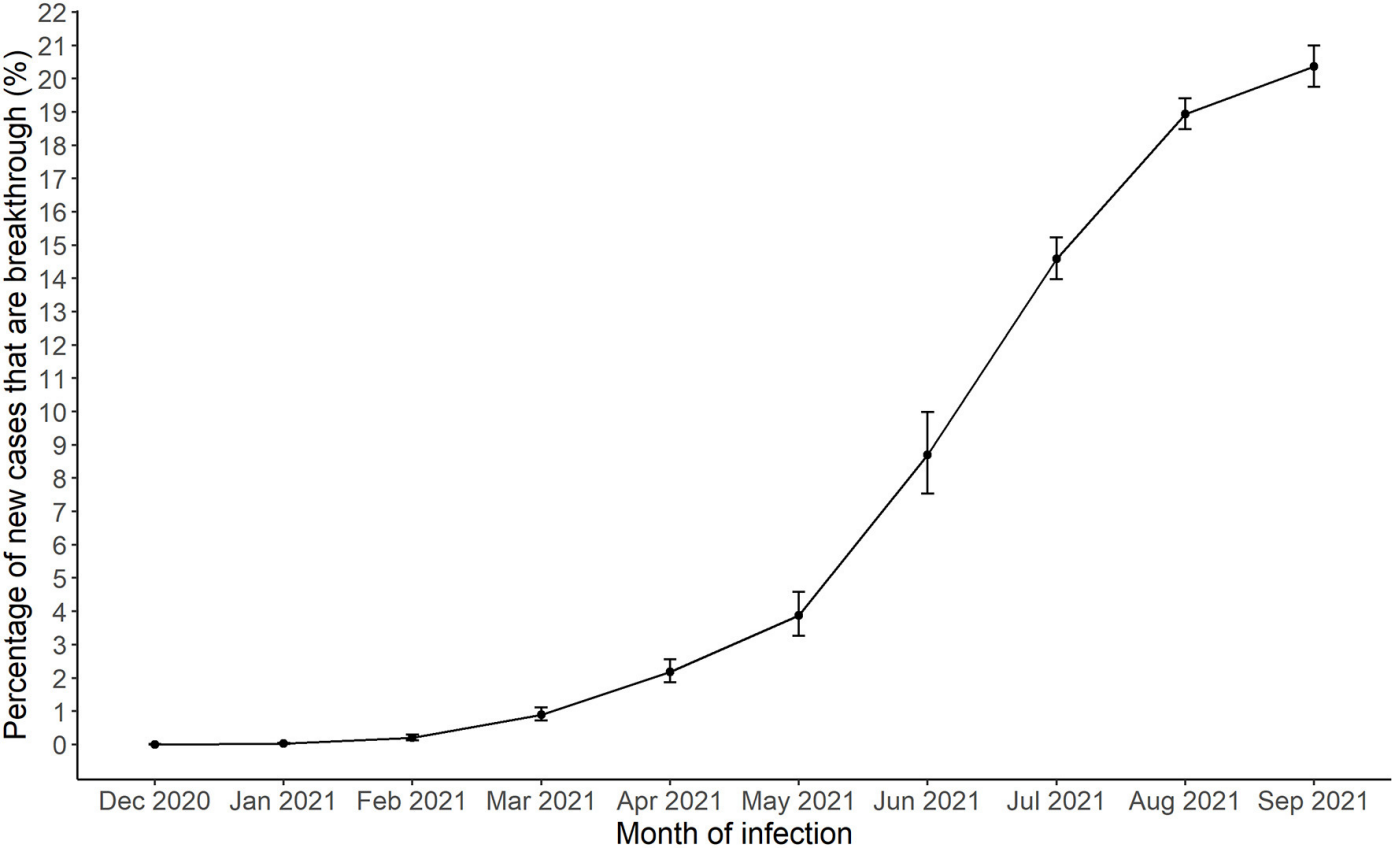
Monthly breakthrough SARS-CoV-2 infections in Harris County Jurisdiction area from December, 2020 to September, 2021.

The percentage of vaccine breakthrough cases increased from 0.03% in January 2021 to 20.0% in September 2021 (Figure 1). Of 1,295 COVID-19 deaths between January and September 2021, 50 were breakthrough cases (3.8%), 24 of which occurred in August 2021 and 13 in September 2021.

### Probability of Breakthrough Infections Over Time

**Figure 2:**
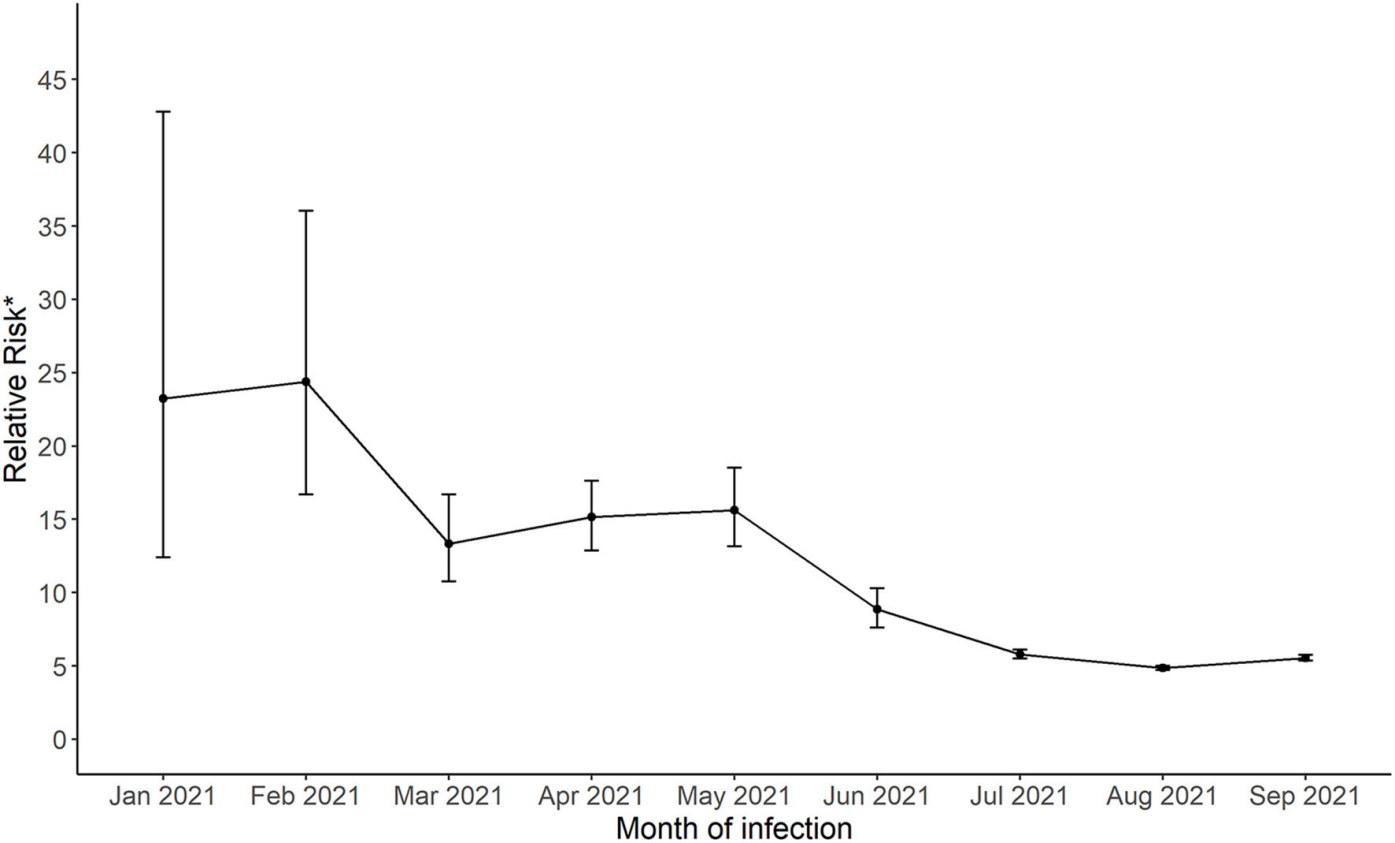
The relative risk of a positive SARS-CoV-2 for not fully vaccinated compared to fully vaccinated groups living in Harris County Jurisdiction area from January, 2021 to September, 2021. $*\text{Relative risk}\;=\;\frac{\text{Pr}\left(\text{positive}\;\text{test}\;|\;\text{not}\;\text{fully}\;\text{vaccinated}\right)}{\mathrm{Pr}\left(\text{positive}\;\text{test}\;|\;\text{fully}\;\text{vaccinated}\right)\;}$.

The estimated probability of having a positive test was significantly lower for the fully vaccinated individuals (monthly range from 0.0002 to 0.005) compared to all others (monthly range 0.002 to 0.022, Supplementary Figure 1) and were highest during months when there was higher community spread in January, July, August and September 2021. The relative risk estimate for January 2021 indicated that unvaccinated/partially vaccinated individuals were at 23.3 times greater risk of having a SARS-CoV-2 infections compared to fully vaccinated individuals (95% confidence interval [CI], 12.4 to 42.8). This estimate significantly decreased to 5.6 (95% CI, 5.3 to 5.8) by September 2021 (Figure 2). The confidence intervals were narrower for later months as the number of fully vaccinated individuals increased. Vaccine effectiveness for infection remained significant throughout the period (all confidence intervals were above 1).

The daily incidence of breakthrough infections per 100,000 (100k) was highest in January, August and September 2021 (Supplementary Figure 2). The results of the Poisson regression are presented in Table 2 and the survival analyses of time until breakthrough infection are in Table 3. The daily incidence per 100k was higher for females (6.84 for females and 6.05 for males; adjusted relative risk [aRR] = 1.14, 95% CI 1.10 to 1.19). The adjusted relative risks were similar in the period before and during when Delta was the dominant strain and was statistically significant during the Delta period (aRR = 1.14 in January-May 2021 and 1.18 in June-September 2021, *P* = 0.02 for the interaction term between gender and time period, Supplementary Table 1). Gender differences were also detected for the time until breakthrough infection in the Cox proportional hazards models (adjusted hazard ratio [aHR] = 1.08, 95% CI 1.04–1.13).

**Table 2:** Daily incidence and relative risk (RR) of SARS-CoV-2 infection incidence among fully vaccinated individuals living in Harris County Jurisdiction area by subgroups.

			**Unadjusted**			**Multivariable model^a^**		
	**Cumulative breakthrough events**	**Daily incidence per 100,000 (95% CI) for January-September 2021**	**RR**	**95% Confidence Interval**	***P***	**aRR**	**95% Confidence Interval**	***P***
Gender								
Female	6,307	6.84 (6.67–7.01)	1.13	1.09–1.17	<0.001	1.14	1.10–1.19	<0.001
Male	4,556	6.05 (5.88–6.23)	Ref.			Ref.		
Age group (years)								
12–19	737	5.86 (5.44–6.29)	1.08	0.99–1.17	0.07	0.97	0.89–1.06	0.57
20–39	3,314	7.53 (7.27–7.79)	1.39	1.32–1.46	<0.001	1.27	1.21–1.35	<0.001
40–59	4,125	6.81 (6.6–7.02)	1.25	1.19–1.32	<0.001	1.16	1.10–1.22	<0.001
80+	352	5.23 (4.69–5.8)	0.96	0.86–1.07	0.50	0.97	0.86–1.09	0.61
60–79	2,516	5.43 (5.22–5.65)	Ref.			Ref.		
Race/ethnicity								
Asian American/Pacific Islander	757	4.7 (4.37–5.05)	0.64	0.60–0.70	<0.001	0.64	0.59–0.69	<0.001
Black	1,230	6.24 (5.9–6.6)	0.86	0.80–0.91	<0.001	0.82	0.77–0.87	<0.001
Hispanic/Latino	3,730	6.76 (6.54–6.98)	0.93	0.89–0.97	<0.001	0.86	0.82–0.90	<0.001
Other	1,061	5.5 (5.17–5.84)	0.75	0.70–0.81	<0.001	0.79	0.74–0.85	<0.001
Unknown	466	5.9 (5.38–6.47)	—					
White	3,800	7.3 (7.07–7.53)	Ref.			Ref.		
Time period fully vaccinated								
6/1/2021–9/30/2021	1,548	5.43 (5.16–5.71)	0.81	0.77–0.85	<0.001	0.47	0.44–0.50	<0.001
12/14/2020–5/31/2021	9,496	6.7 (6.57–6.84)	Ref.			Ref.		
Vaccine trade name +/– booster								
Moderna + booster	34	1.21 (0.84–1.69)	0.25	0.17–0.34	<0.001	0.28	0.19–0.38	<0.001
Janssen	1,371	9.79 (9.28–10.33)	2.00	1.88–2.13	<0.001	2.00	1.87–2.13	<0.001
Janssen + booster	10	7.91 (3.79–14.55)	1.62	0.81–2.84	0.13	1.66	0.83–2.92	0.11
Pfizer	6,684	7.62 (7.44–7.8)	1.56	1.49–1.63	<0.001	1.60	1.53–1.67	<0.001
Pfizer + booster	59	0.89 (0.68–1.15)	0.18	0.14–0.23	<0.001	0.19	0.14–0.24	<0.001
Moderna	2,840	4.89 (4.72–5.08)	Ref.			Ref.		
R(t) >1	—	—	0.97	0.92–1.01	0.16	1.09	1.03–1.15	0.001

a *Multivariable models are adjusted for all other variables in the table*.

**Table 3:** Cox proportional hazards model for time until breakthrough infection among subgroups of 1,272,611 fully vaccinated residents in Harris County Jurisdiction.

	**Unadjusted**			**Multivariable model^a^**		
	**Hazard Ratio**	**95% Confidence Interval**	***P***	**Hazard Ratio**	**95% Confidence Interval**	***P***
Gender						
Female	1.08	1.04–1.13	<0.001	1.08	1.04–1.13	<0.001
Male	Ref.			Ref.		
Age group (years)						
12–19	1.90	1.75–2.06	<0.001	1.55	1.42–1.69	<0.001
20–39	1.74	1.65–1.83	<0.001	1.64	1.55–1.73	<0.001
40–59	1.45	1.38–1.53	<0.001	1.37	1.30–1.45	<0.001
80+	0.90	0.81–1.01	0.07	0.89	0.79–1.00	0.05
60–79	Ref.			Ref.		
Race/ethnicity						
Asian American/Pacific Islander	0.66	0.61–0.71	<0.001	0.63	0.58–0.68	<0.001
Black	0.91	0.85–0.97	0.003	0.84	0.78–0.89	<0.001
Hispanic/Latino	1.06	1.01–1.11	0.01	0.94	0.89–0.98	0.005
Other^b^	0.78	0.73–0.84	<0.001	0.78	0.73–0.84	<0.001
White	Ref.			Ref.		
Vaccine trade name +/– booster						
Moderna + booster	0.19	0.13–0.26	<0.001	0.21	0.15–0.29	<0.001
Janssen	2.08	1.95–2.22	<0.001	1.87	1.75–2.00	<0.001
Janssen + booster	1.57	0.84–2.92	0.15	1.47	0.79–2.73	0.22
Pfizer	1.69	1.62–1.76	<0.001	1.65	1.58–1.73	<0.001
Pfizer + booster	0.14	0.11–0.18	<0.001	0.14	0.11–0.19	<0.001
Moderna	Ref.			Ref.		
7-day moving average of new daily infections at the time of being fully vaccinated (per 1000)	0.97	0.92–1.03	0.34	1.17	1.11–1.24	<0.001
R(t) estimate on day of being fully vaccinated (per 0.1 units)	1.10	1.08–1.12	<0.001	1.08	1.06–1.10	<0.001

a *Multivariable models are adjusted for all other variables in the table*.

b *The American Indian/Alaskan Native and Multi-racial groups were included in the “other” category. Individuals with unknown race/ethnicity were removed from the statistical models. Multivariable models are adjusted for all other variables in the table*.

The daily incidence of breakthrough infections was highest in the 20–39-year-old group (7.53 per 100k) and lowest in the 80+ year-old group (5.23 per 100k). In the multivariable Poisson models, compared to the 60–79-year group, the 20–39-year-old and 40–59-year-old groups had significantly higher rates of infections (20–39 year aRR = 1.27, 95% CI 1.21 to 1.35; 40–59 year aRR = 1.16, 95% CI 1.10 to 1.22). The 12–59 year-old groups had 37% to 64% higher hazard compared to the 60–79 year group (Table 3; Supplementary Figure 3).

Whites had the highest daily incidence of breakthrough infections (7.30 per 100k), followed by Hispanics (6.76 per 100k), Blacks (6.24 per 100k), and Asians had the lowest (4.70 per 100k). Compared to the White group and after adjustment for gender, age, vaccine manufacturer with booster status, and community spread, Asians had significantly lower incidence rates (aRR = 0.64, 95% CI 0.59 to 0.69) and lower hazard ratios (aHR = 0.63, 95% CI 0.58 to 0.68; Supplementary Figure 4). Whites had significantly higher aRR, particularly during the Delta wave (*P* = 0.01 for interaction between race/ethnicity and time period, Supplementary Table 1).

The Janssen COVID-19 vaccine had the highest daily incidence of breakthrough infections (no booster: 9.79 per 100k; at least one booster: 7.91 per 100k) and the Pfizer COVID-19 vaccine with at least one booster had the lowest (0.89 per 100k) followed by the Moderna COVID-19 vaccine with at least one booster (1.21 per 100k). The Pfizer COVID-19 vaccine without booster had similar daily incidence to the Janssen with at least one booster (7.62 per 100k). Compared to Moderna, higher infection rates were observed for Janssen without booster (aRR = 2.00, 95% CI = 1.87 to 2.13) and Pfizer without booster (aRR = 1.60, 95% CI = 1.53 to 1.67), lower infection for Pfizer + booster (aRR = 0.19, 95% CI = 0.14 to 0.24), and lower infection rates for Moderna + booster (aRR = 0.28, 95% CI = 0.19 to 0.38), after adjustment for gender, age, race/ethnicity, and community spread. Similar trends were observed for time until breakthrough infections, adjusted for demographics and community spread (Table 3; Supplementary Figure 5).

The R(t) estimate, a measure of the whether infections are increasing [R(t) > 1], was associated with a 9% increase in infections (aRR = 1.09, 95% CI = 1.03–1.15) and the hazard ratio was 118% higher for every 0.1 unit increase in the R(t) estimate (aHR = 2.18, 95% CI 1.80 to 2.64, Table 3).

## Discussion

In this investigation using community-based data in a large diverse metropolitan area, we show that the SARS-CoV-2 infection rate, including non-severe COVID-19, was significantly higher among individuals not fully vaccinated compared to those fully vaccinated. However, that risk attenuated over the first half of 2021, from 23 times higher for unvaccinated in January 2021 to 6 times higher in September 2021. A hypothesis for the breakthrough trend may be the influence of the delta variant which became more prevalent in Texas in June through September and has been estimated to be significantly more infectious (7, 9). It is also plausible that the effectiveness of COVID-19 vaccines is waning over time.

The effectiveness of COVID-19 vaccines against the delta variant for symptomatic disease has been estimated at 88% for the Pfizer vaccine and 49% among individuals 18–64 years of age that received either Pfizer or AstraZeneca vaccines, an overall reduction compared to previous variants (10, 11). It is plausible that the increase in the number of breakthrough infections is at least partly a result of this reduced effectiveness. There may be confounding between the possibilities that the vaccines are potentially losing efficacy over time or that they are less protective against the delta variant.

In contrast with subgroup analyses conducted in the Phase III randomized trials of the vaccines, our analysis detected heterogeneity in the risk of breakthrough infections by gender, age, and race/ethnicity. Our study builds upon previous work by evaluating estimates of infection risk, stratifying the analysis according to the time period since delta became predominant in our region, and adjusting for community spread. Females had a 14% higher risk of breakthrough infections, and 8% increase in hazard for time until breakthrough. In Harris county, 54% of the fully vaccinated were women and in our study, women represented 58% of breakthrough infections before June, and 56% afterwards. In a recent report including 10,262 breakthrough infections in 46 U.S. states through April 2021, the CDC indicated that 63% of cases were female, which reflected the proportion of women fully vaccinated at that time (12). In a similar period, a study including 1,497 fully vaccinated health care workers in Israel observed 39 pre-delta breakthrough cases, of which 64% were women, a similar percentage in the control group (67%) (4). Further studies of differences by gender, that control for any exposure differences by gender (e.g. work and family) and for testing rates (e.g. with a fixed testing schedule), are warranted.

In our study, older adults (60+) had the lowest risk of breakthrough infections. They were also the first age group to become eligible for booster vaccines. The 20–39-year-old group had between 21% and 35% increased risk for a breakthrough infection, compared to 60–79-year-olds. The 40–59 year group also had an increased risk. This may reflect behavioral differences in terms of exposure and non-pharmaceutical interventions (e.g. mask wearing, social distancing) during this period. Similarly, the differences in breakthrough infections by race/ethnicity have not been studied previously and may be a result of behavioral and cultural differences.

Finally, we observed a modest, but significant, difference in risk of breakthrough infections between Moderna and Pfizer, with Moderna appearing to be the most protective against variants in Harris County between December and September 2021. The addition of a booster to either mRNA vaccine led to significantly decreased risk of infection. In addition, consistent with randomized trials showing relatively lower effectiveness of the Janssen vaccine, we observed higher risk of breakthrough infections among individuals who received the Janssen COVID-19 vaccine, compared to those receiving either Moderna and Pfizer. The cumulative events started to notably increase after about 3 months post full vaccination for the Janssen vaccine, and the other two curves diverged more about 6 months post full vaccination. Due to the differential roll-out of the vaccines, this time period may coincide with the Delta surge (Supplementary Figure 5). Additionally, Pfizer and Moderna vaccines were administered in the earliest phases of vaccine rollout to those with significant exposure (health care workers) and older residents. Thus, the increase observed in the Pfizer curve may reflect those people at highest risk who were then exposed during the delta surge (six months after being fully vaccinated). The lower Janssen effectiveness does not fully explain the gender, age, and race/ethnicity differences we observed.

This study had some important limitations. First, the matching of case and vaccination data used name and date of birth, but may have missed matches if this information was either incomplete or incorrect. For example, name changes during our observation period may not be reflected in the data and out-of-state vaccinations may not have been shared between states. These missed matches for those with a positive test are classified as unvaccinated if they were not identified in the vaccination data. Second, there may be a substantial testing bias if the likelihood of getting a NAAT test for SARS-CoV-2 differs by vaccination status or by other factors such as socio-economic status, exposure, and occupation. Third, we do not know whether infected individuals had severe symptoms or were hospitalized for COVID-19, and thus were not able assess the severity of breakthrough infections. Fourth, the definition of partial breakthroughs included individuals who were vaccinated, but not fully vaccinated, and received a positive test in days 0–13 post vaccination. There are possible temporality issues with this definition given that a positive test is most likely 3–5 days after infection and an individual may not have the opportunity to mount a response. A different choice for the starting time for defining partial breakthroughs may also have limitations. However, partial breakthroughs were only 4% of all positive tests, and changing the definition would only affect a fraction of these.

The current study suggests that although the probability of breakthrough infections has increased in the first half of 2021, Janssen, Pfizer, and Moderna vaccines are significantly effective against the Delta variant for not only severe disease but also reducing infection. Various correlates of breakthrough infection should be confirmed in future studies to help understand risk differences among subgroups and to guide vaccine series recommendations. Testing of both unvaccinated and vaccinated individuals should be increased to capture asymptomatic/mild infections that may lead to increased community spread. The data indicate that increased vaccine booster uptake would help decrease new infections.

## Data Availability Statement

The data analyzed in this study is subject to the following licenses/restrictions: County-level data on COVID-19 infections are publicly available through the Texas Department of State Health Services. Vaccination data is not publicly available. The University of Texas Health Science Center at Houston (UTHealth) School of Public Health has a data use agreement with Harris County Public Health to use protected health information data and have been collaborating since July 2020 on the pandemic response. Requests to access these datasets should be directed to J-MY, jose-miguel.yamal@uth.tmc.edu.

## Ethics Statement

The studies involving human participants were reviewed and approved by the Committees for the Protection of Human Subjects of University of Texas Health Science Center at Houston. Written informed consent from the participants' legal guardian/next of kin was not required to participate in this study in accordance with the national legislation and the institutional requirements.

## Author Contributions

J-MY, SA, MW, LL-N, EB, and MdOO contributed to the conception and design of the study. J-MY wrote the first draft of the manuscript. SA, MW, and MdOO wrote sections of the manuscript. SA, AR, and EB managed the database. J-MY, SA, MW, LL-N, and AR performed the statistical analyses. All authors contributed to manuscript revision, read, and approved the submitted version.

## Funding

This work was supported by a contract between Harris County Public Health and University of Texas Health Science Center at Houston School of Public Health as part of our ongoing collaboration on the pandemic response.

## Conflict of Interest

The authors declare that the research was conducted in the absence of any commercial or financial relationships that could be construed as a potential conflict of interest.

## Publisher's Note

All claims expressed in this article are solely those of the authors and do not necessarily represent those of their affiliated organizations, or those of the publisher, the editors and the reviewers. Any product that may be evaluated in this article, or claim that may be made by its manufacturer, is not guaranteed or endorsed by the publisher.
