# Supplementary material for: Trends and Correlates of Breakthrough Infections With SARS-CoV-2
Source: Front Public Health. 2022 May 10;10:856532. doi: 10.3389/fpubh.2022.856532 (PMC9127615; doi:10.3389/fpubh.2022.856532)
Supplement: Supplementary file 1 [file Data_Sheet_1.PDF]

Supplementary Appendix

Table of Contents

Supplemental Figure 1. Estimated probability of having a positive SARS-CoV2 test given fully vaccinated (solid line) and not fully vaccinated (dashed line).......**Error! Bookmark not defined.**

Supplemental Figure 2. Monthly Incidence density of a breakthrough....... **Error! Bookmark not defined.**

Supplemental Table 1. Relative risk (RR) of SARS-CoV2 infection incidence among fully vaccinated individuals living in Harris County Jurisdiction area by subgroups and by time period 3

Supplemental Table 2. Daily incidence and relative risk (RR) of SARS-CoV2 infection incidence among fully vaccinated individuals living in Harris County Jurisdiction area by vaccine series completed and booster vaccine combination. 5

Supplemental Figure 3. Cumulative event rates for breakthrough infections by age group.. **Error! Bookmark not defined.**

Supplemental Figure 4. Cumulative event rates for breakthrough infections by race/ethnicity group. ....**Error! Bookmark not defined.**

Supplemental Figure 5. Cumulative event rates for breakthrough infections by vaccine +/- booster group. ....**Error! Bookmark not defined.**

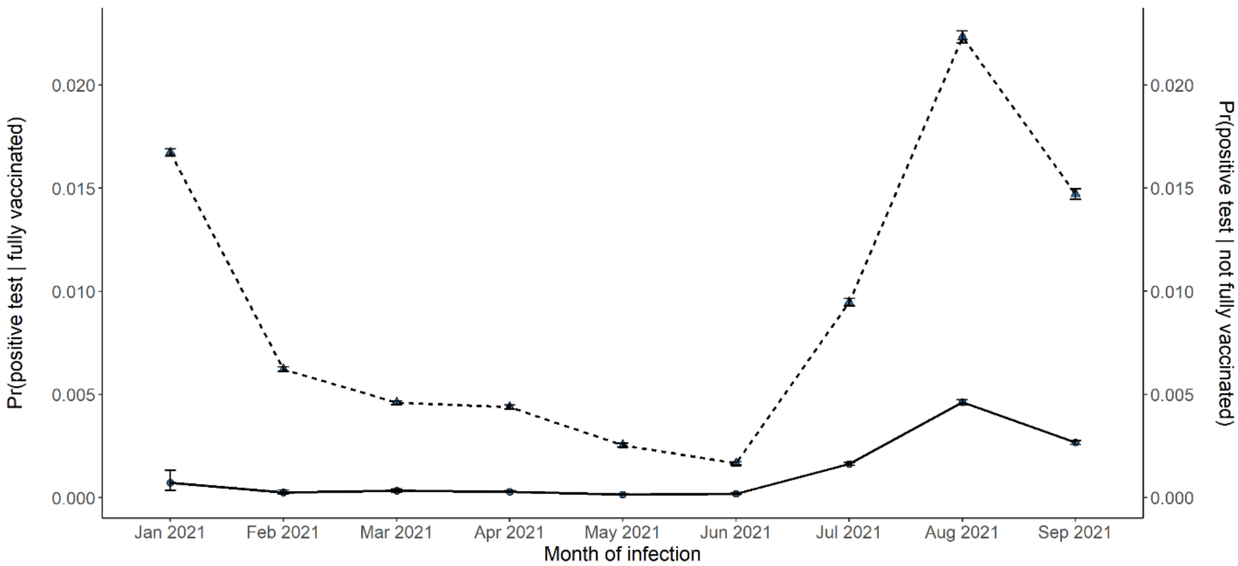

Supplemental Figure 1. Estimated probability of having a positive SARS-CoV2 test given fully vaccinated (solid line) and not fully vaccinated (dashed line)

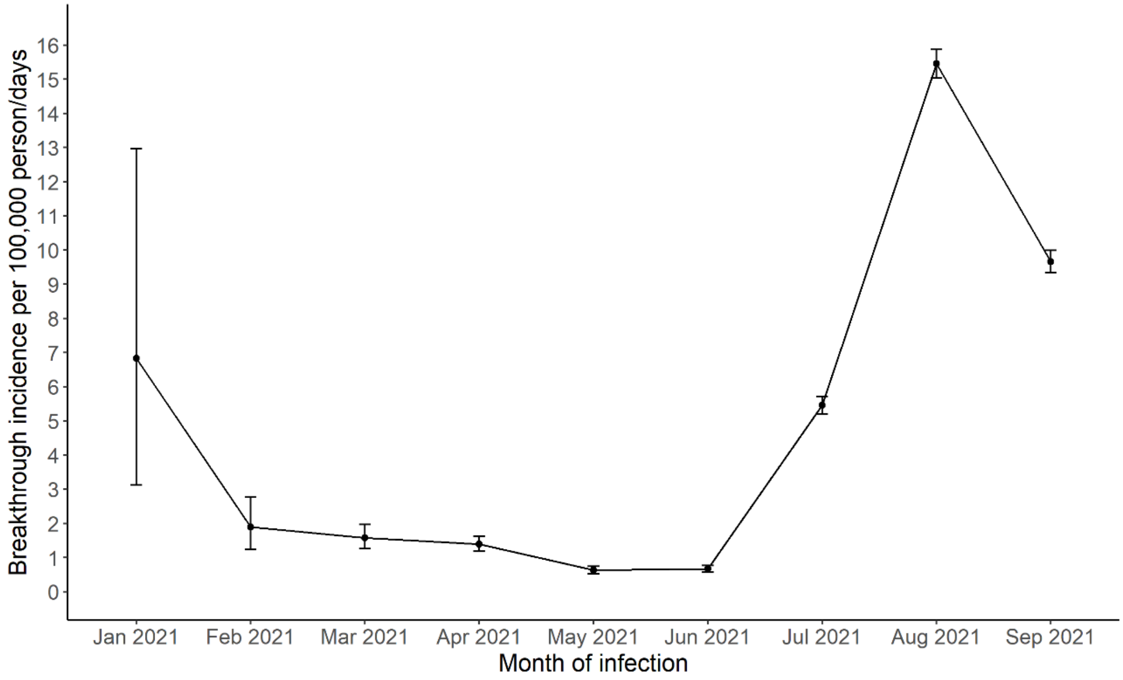

Supplemental Figure 2. Monthly Incidence density of a breakthrough

Supplemental Table 1. Relative risk (RR) of SARS-CoV2 infection incidence among fully vaccinated individuals living in Harris County Jurisdiction area by subgroups and by time period

Subgroup	P value for interaction between time period and each subgroup	January-May 2021				June-September 2021			
		Unadjusted RR	*P* Value	Multivariable model aRR	*P* value	Unadjusted RR	*P* Value	Multivariable model aRR	*P* Value
Gender[a]	0.02								
Female		1.18	0.11	1.14	0.23	1.16	<0.001	1.18	<0.001
Male		Ref.		Ref.		Ref.		Ref.	
Age group (years)	0.46								
12-19		0.60	0.26	0.55	0.20	0.85	<0.001	0.69	<0.001
20-39		1.26	0.09	1.14	0.38	1.20	<0.001	1.09	0.002
40-59		1.36	0.01	1.26	0.07	1.13	<0.001	1.04	0.11
80+		1.67	0.01	1.52	0.04	0.97	0.66	0.98	0.72
60-79		Ref.		Ref.		Ref.		Ref.	
Race/ethnicity[b]	0.01								
Asian American/ Pacific Islander		0.35	<0.001	0.38	<0.001	0.64	<0.001	0.65	<0.001
Black		1.15	0.36	1.08	0.63	0.82	<0.001	0.78	<0.001
Hispanic/Latino		1.02	0.88	1.04	0.74	0.85	<0.001	0.81	<0.001
Other		0.93	0.65	0.90	0.53	0.74	<0.001	0.76	<0.001
Unknown		—		—		—		—	
White		Ref.		Ref.		Ref.		Ref.	
Vaccine name +/- booster	0.58								
Moderna + booster		0	>0.99	0	0.98	0.32	<0.001	0.35	<0.001
Janssen		4.25	<0.001	4.37	<0.001	1.92	<0.001	1.98	<0.001
Janssen + booster		5.11	0.10	5.37	0.10	1.54	0.20	1.65	0.13
Pfizer		2.20	<0.001	2.16	<0.001	1.48	<0.001	1.53	<0.001
Pfizer + booster		0.13	0.01	0.11	0.002	0.22	<0.001	0.22	<0.001
Moderna		Ref.		Ref.		Ref.		Ref.	
R(t) >1	0.16	1.22	0.10	1.08	0.58	0.90	<0.001	0.78	<0.001

Abbreviations: aRR, adjusted relative risk (exponentiated coefficient from multivariable Poisson model); CI, confidence interval; Ref, reference category; RR, relative risk (exponentiated Poisson model coefficient)

[a]There were 10 breakthrough events among "other" gender (11.59 daily incidence per 100,000; 95% CI 5.56 to 21.32) and 171 breakthrough events among unknown gender groups (6.42 daily incidence per 100,000; 95% CI 5.49 to 7.45).
[b]In the Poisson models, the American Indian/Alaskan Native and Multi-racial groups were included in the "other" category. Individuals with unknown race/ethnicity were removed from the statistical models. Multivariable models are adjusted for all other variables in the table.

Supplemental Table 2. Daily incidence and unadjusted relative risk (RR) of SARS-CoV2 infection incidence among fully vaccinated individuals living in Harris County Jurisdiction area by vaccine series completed and booster vaccine combination.

	Cumulative breakthrough events	Daily incidence per 100,000 (95% CI) for January-September 2021	Unadjusted RR	95% Confidence Interval	*P*
**Vaccine trade name +/- booster combination**					
Janssen	1371	9.79 (9.28–10.33)	7.95	5.60–11.28	<0.001
Janssen + Janssen (B)	7	13.09 (5.26–26.97)	10.62	4.69–24.07	<0.001
Janssen + Moderna (B)	2	7.02 (0.85–25.36)	5.70	1.37–23.77	0.02
Janssen + Pfizer (B)	1	2.25 (0.06–12.53)	1.82	0.25–13.35	0.55
Moderna	2840	4.89 (4.72–5.08)	3.97	2.80–5.63	<0.001
Moderna + Janssen (B)	-	-	0.00	0.00–Inf	>0.99
Moderna + Pfizer (B)	2	1.03 (0.12–3.71)	0.83	0.20–3.48	0.80
Pfizer	6684	7.62 (7.44–7.80)	6.18	4.37–8.75	<0.001
Pfizer + Janssen (B)			0.00	0.00–Inf	>0.99
Pfizer + Moderna (B)	1	0.9 (0.02–5.00)	0.73	0.10–5.33	0.75
Pfizer + Pfizer (B)	58	0.89 (0.68–1.16)	0.73	0.47–1.12	0.15
Moderna + Moderna (B)	32	1.23 (0.84–1.74)	Ref.		

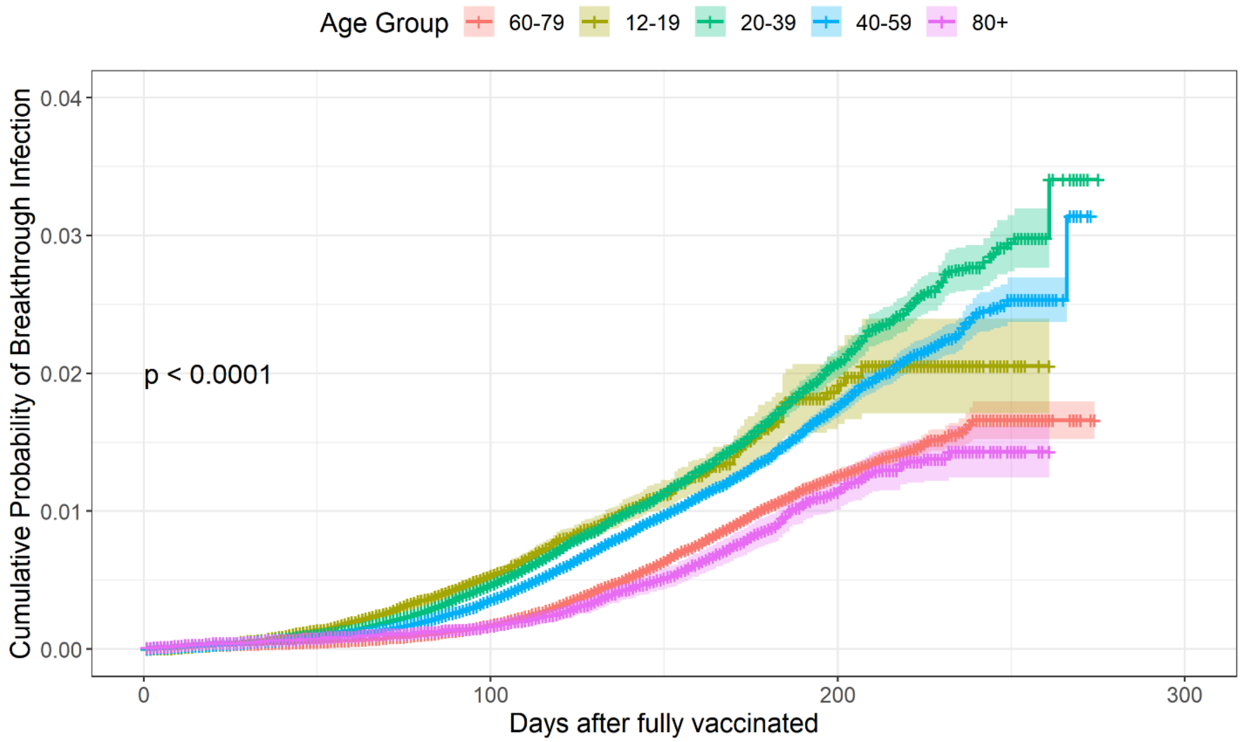

Supplemental Figure 3. Cumulative event rates for breakthrough infections by age group.

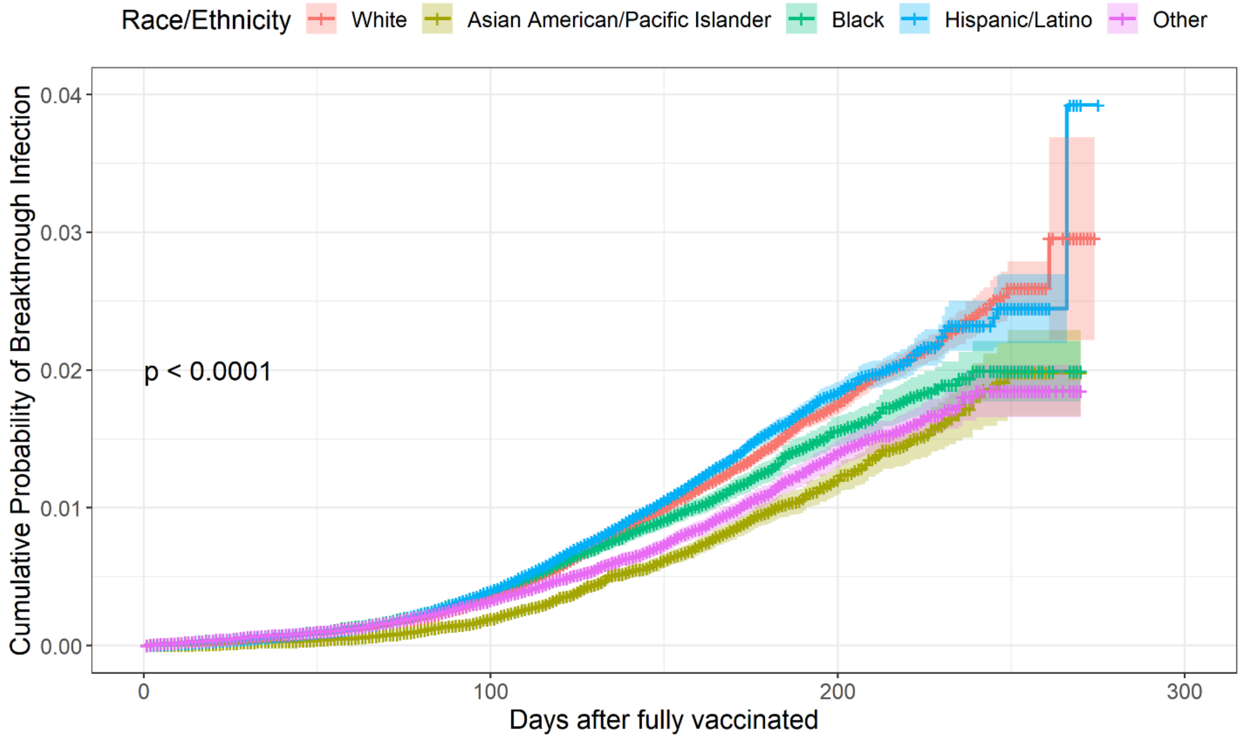

Supplemental Figure 4. Cumulative event rates for breakthrough infections by race/ethnicity group.

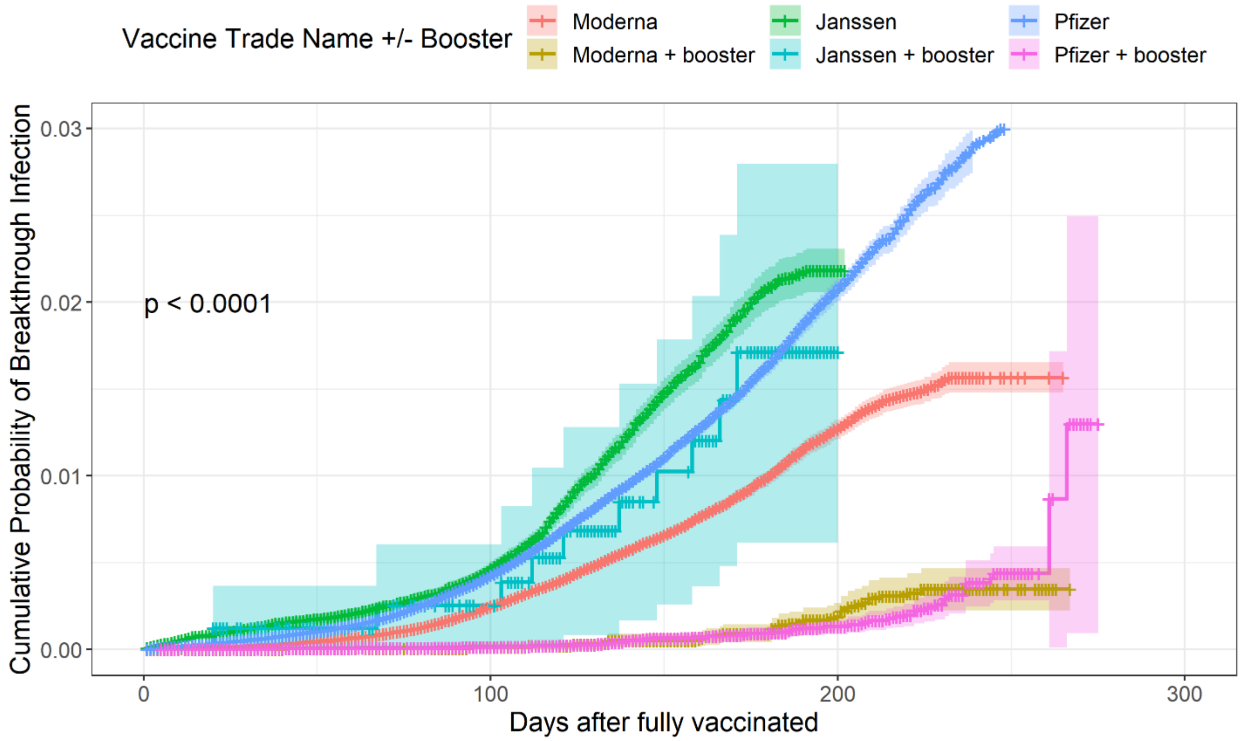

Supplemental Figure 5. Cumulative event rates for breakthrough infections by vaccine +/- booster group.
